# Lifestyle Factors and Cognitive Ageing: Variation across Ability and Lifestyle Domains

**DOI:** 10.1155/2012/143595

**Published:** 2012-11-04

**Authors:** Alan J. Gow, Allison A. M. Bielak, Denis Gerstorf

**Affiliations:** ^1^Centre for Cognitive Ageing and Cognitive Epidemiology, Department of Psychology, University of Edinburgh, Edinburgh EH8 9JZ, UK; ^2^Department of Human Development and Family Studies, Colorado State University, Fort Collins, CO 80523-1570, USA; ^3^Institute of Psychology, Humboldt University of Berlin, 12489 Berlin, Germany; ^4^German Institute for Economic Research (DIW), 10117 Berlin, Germany

Cognitive abilities change with age. However, as there are marked individual differences in the timing and trajectory of these changes [1, 2], it is a research priority to discover factors that contribute to these differences. Lifestyle factors, particularly those which are malleable across the life course, may be highly informative for the development of interventions to reduce or delay age-related cognitive decline. Factors of interest, to name only a few, include aspects of social, leisure, and physical activity; personality; social networks, support and relationships; and health behaviours [3]. The purpose of this special issue was to bring together research that addresses the associations between cognition in older adulthood and these various factors, thereby allowing overarching themes, trends, and conclusions to emerge.

Efforts to identify lifestyle factors that are associated with cognitive ageing are challenged by a number of methodological issues. For example, conclusions are often limited by short periods of follow-up or an assessment of a narrow range of cognitive abilities. In addition, lifestyle factors are often considered in isolation, which, although convenient, may not accurately reflect the way in which individuals actually experience these factors. Approaching the issue from a multivariate perspective might allow the identification of which factors have a more prominent association with cognition over and above other influences. Another concern is the difficulty of distinguishing between alternative explanations for a given lifestyle factor's association with cognitive ability: does the lifestyle factor lead to differential preservation, that is, does it actually predict subsequent differential cognitive change? Alternatively, is the association the result of preserved differentiation, where the factor is associated with individual differences in baseline performance and with the maintenance of these differences over time, but not with further differential change [4–6]?

Each of the papers in this special issue describes an attempt to tackle one or more of the unknowns about associations between lifestyle factors and cognition, including the consideration of neurological factors underlying some of the observed effects. Although no single study can address all pertinent issues, those included often combined a long period of follow-up in old age with a diverse assessment of cognitive abilities. One set of studies sampled a wide range of lifestyle factors simultaneously so as to compare their effects with one another, and another suite of papers considered a range of lifestyle factors across distinct samples. A number of the papers approached the differential preservation/preserved differentiation conundrum by considering lifestyle effects on both cognitive ability level and cognitive change across time. The papers in this special issue are broadly arranged into four themes:

- mental health and health behaviours,
- social activity and networks,
- cognitive engagement, and
- neurological factors.

The previously mentioned issues are spread throughout these four themes, highlighting that researchers are cognizant of the need to address these critical issues across lifestyle domains. We first summarize each of the papers within these domains before providing an overall conclusion from this suite of papers.

**Mental Health and Health Behaviours**

Firstly, E. L. Mortensen et al. examined whether hostility and depression shape developmental trajectories of cognitive abilities across adulthood. Under the premise that these personality factors are linked with various aspects of stress, the authors applied growth curve modelling to cognitive data obtained from a sample tested every ten years over a period of 30 years. After adjusting for sociodemographic variables and a wide range of lifestyle factors (e.g., blood pressure, smoking, physical activity, and obesity, as well as blood samples of serum cholesterol, triglycerides, and insulin), results revealed that depressive traits and hostility were indeed associated with cognitive ability level, but there was no prediction of differential change.

In D. Cadar et al., the authors investigated the unique and combined effects of various health behaviours measured in midlife—smoking, physical activity, and nutrition—on later cognitive ability. Dietary choices and physical activity were independently associated with cognitive change, particularly in the memory and perceptual speed domains.

Though M. Lindwall et al. considered only one of these same health behaviours—physical activity—they addressed the associations using a coordinated analytical approach across four distinct longitudinal samples (two further papers from this group focusing on alternative lifestyle factors appear later in this special issue). Accounting for expected age-related declines, changes in physical activity were associated with concomitant changes in reasoning and fluency.

**Social Activity and Networks**

Three papers in this special issue consider associations between social lifestyle factors and cognitive ageing. In the first, L. C. Giles et al. examined whether a particular aspect of an individual's social network was linked to cognitive ageing in old and very old age. Of the different types of social networks assessed—children and other family, friends, and confidant—it was particularly the effect of an individual's network of friends (number, personal contact, and phone contact) that was beneficial for maintaining episodic memory across the 15-year follow-up.

C. L. Brown et al. also focused on associations between social activity and cognition, and represents the second paper using a coordinated analysis of four longitudinal samples. Across studies and multiple cognitive domains (reasoning, memory, fluency, and semantic knowledge), the authors consistently observed associations with level for most of the abilities assessed, but there was little evidence that social activities predicted differential cognitive change.

Finally in this section, S. M. Sisco and M. Marsiske took a broader view of lifestyle influences on cognition by examining whether neighbourhood-level socioeconomic position was associated with cognitive ability, and whether this association was stronger for certain abilities than for others. The authors reported that vocabulary was the only domain uniquely affected by neighbourhood factors, suggesting social acculturation effects of one's environment. However, they also acknowledged possible alternative interpretations, including selection effects and the lingering influence of the socioeconomic position of one's childhood neighbourhood.

**Cognitive Engagement**

The next group of studies examined the effect of cognitive engagement, stimulation, or activity. Firstly, J. M. Parisi et al. investigated cross-sectional associations between education and a range of cognitive abilities (reading ability, processing speed, and memory), while considering the potential mediating role of a variety of lifestyle factors, including intellectual, social, physical, creative, and passive activities. Although strong inferences cannot be drawn from cross-sectional mediation models, both separate and conjoint analyses revealed intellectual activities as the strongest mediator between education and cognition.

S. von Stumm took a different perspective on the activity-cognition issue by comparing how activity engagement and its link with cognition might differ from that between the predisposition to engage in these activities (investment trait) and cognition. Interestingly, the predisposition to engage in activity, but not cognitive activity itself, was related to baseline cognition, though neither factor mediated the association between age and cognitive ability.

 In the last of the papers combining the results from four samples, M. B. Mitchell et al. reported associations between cognitive activity and cognitive ability across a number of domains. Though there were significant baseline associations, there was no evidence of baseline activity predicting subsequent cognitive change. However, associations between changes in activity and changes in cognitive ability were apparent, once again highlighting the challenges of examining temporal precedence, even with longitudinal data.

**Neurological Factors**

The remaining two papers moved beyond describing associations between lifestyle factors and cognitive ageing to trying to elucidate potential neurological factors shaping such linkages. In B. D. James et al., associations between social engagement and structural parameters from MR brain imaging were examined. Higher social engagement was associated with larger total brain and grey matter volumes, though not white matter volume. Such findings are interesting, particularly because lifestyle factors are proposed as cognitively protective. It is important to describe and better understand the actual physical effects such lifestyle factors might have on the ageing brain.

Similarly, T. D. Verstynen et al. used structural MR imaging to test whether cardiorespiratory fitness was associated with cognitive flexibility and attentional control. The results suggested that cardiorespiratory fitness predicted cognitive flexibility via larger greater grey matter volume in the dorsal striatum.

**Conclusions**

The papers included in this special issue considered a number of lifestyle factors which have been proposed as cognitively protective. Indeed, the results of some papers are in accordance with that proposal, but not all. Rather, what might be more informative for our understanding about this complex relationship is what generalities appeared across the suite of papers, at the same time as acknowledging persistent differences by cognitive domain and lifestyle factor.

The simultaneous examination of lifestyle effects on both cognitive ability level and longitudinal cognitive change in many of the papers suggests that concurrent associations of a factor with cognitive ability do not necessarily generalize to significant associations with cognitive change. The repeated demonstration of this phenomenon across multiple areas (social networks, physical, social, and cognitive activity, depression, diet, and smoking) is relevant to the debate between preserved differentiation versus differential preservation. If a factor is not associated with differential cognitive change, is the factor still relevant to the eventual goal of promoting healthy cognitive ageing? These findings can have implications for those interested in translating such observational findings into interventions aimed at reducing or delaying age-related cognitive decline. Where there are few associations with cognitive change, then interventions may be less likely to succeed. However, associations between lifestyle factors and cognitive ability level might suggest the potential to increase cognitive ability *level* across the lifespan via lifestyle-based interventions. An increase in level of cognitive ability would still be of benefit because (everything else being equal) it would increase the time before reaching some defined threshold for impairment.

 At the same time as noting commonalities, we would be remiss to ignore the persistent variation that seems to exist throughout this special issue. The results of the present studies varied extensively by the investigated lifestyle factor, including activity domain, and the cognitive domain considered. It may be that the associations between each lifestyle and cognitive domain are unique and subject to variation, or it may be that they are in fact similar along various threads or facets that we simply have not yet identified. Regardless, there are clearly many additional questions to be investigated. To aid in this search, we suggest that studies of cognitive ageing include a diverse assessment of cognitive ability across a number of domains. Such detailed assessments are often lacking from studies in this literature, and those utilising a single broad, often basic screening measure (such as the Mini-Mental State Examination [7]) are not uncommon. Other approaches worthy of greater usage would be the combining of disparate studies in coordinated analyses, as demonstrated by several papers in this special issue, potentially allowing constructive replication of the effects investigated [8]. Analyses which also involve the simultaneous consideration of multiple lifestyle factors might more realistically examine the ways in which individuals participate in a range of somewhat interrelated behaviours, and how these combinations are associated with cognitive ability in older adulthood.

The search for malleable determinants of cognitive ageing is important, even more so with the trend towards increasingly older populations. The individual variation observed in cognitive ageing trajectories is a tantalising hint that factors which influence this do exist, though the identification of these likely small effects has been difficult. In closing, we note that one of the next steps the field could substantially benefit from would be to move from examining between-person associations to a thorough investigation of within-person associations [9]. To do so, however, studies with more frequent and more closely spaced multi-domain measurements are needed. It is only then that we can examine whether and how lifestyle factors and cognitive ageing indeed go hand in hand at the individual (rather than the population) level.
